# Tricortical iliac crest allograft with anterolateral single rod screw instrumentation in the treatment of thoracic and lumbar spinal tuberculosis

**DOI:** 10.1038/s41598-020-70007-z

**Published:** 2020-08-03

**Authors:** Yanping Zeng, Yong Fan, Fei Luo, Tianyong Hou, Fei Dai, Jianzhong Xu, Zehua Zhang

**Affiliations:** 1 Department of Orthopaedics, Southwest Hospital, Chongqing, 400038 China; 2 Department of Orthopaedics, Xi’an Honghui Hospital, Xi’an, China

**Keywords:** Tuberculosis, Spinal cord diseases

## Abstract

To assess the effectiveness of tricortical iliac crest allografts with anterolateral instrumentation after single-stage surgery for thoracic and lumbar spinal tuberculosis (TB). Fifty-six patients with thoracic and lumbar spinal TB underwent single-stage anterior radical debridement, interbody fusion with tricortical iliac crest allografts and anterolateral single rod instrumentation. All patients were given 18 months of antituberculosis chemotherapy. The patients were followed up regularly, and their clinical manifestations, roentgenogram results, erythrocyte sedimentation rate (ESR) and liver function test were the results to be concerned. Radiographs were analysed before surgery, immediately after surgery, and at the final follow-up examination. Mean follow-up period was 37.5 months in 52 patients, and 4 patients were lost to follow-up. No patients had superficial wound infections, and all the incisions healed within 2 weeks. No graft fracture, collapse, or sliding was observed. The average bony fusion time was 10.6 months. Bony fusion was observed in all 52 patients within 18 months. The average degrees of kyphotic correction loss for thoracic and lumbar spine were 6.71° and 2.78° respectively. Although it took a long time to achieve solid fusion, tricortical iliac crest allografts were found to be convenient and safe to be used in spinal TB surgery. They may be effective options for interbody fusion, deformity correction and correction maintenance with anterolateral single rod instrumentation.

## Introduction

China accounts for nearly 11% of the TB burden worldwide, second only to India in the highest number of TB patients^1^. The World health organization (WHO) declared TB a worldwide emergency in 1993. Since then, spinal TB, a common form of extrapulmonary TB, has resurged in China after decades of low incidence. According to Yu Pang’s study, the most frequent forms of extrapulmonary TB observed were skeletal TB (41.1%), and spinal TB accounted for half of the skeletal TB infections^2,3^.

Antituberculosis chemotherapy is still the cornerstone in the management of skeletal TB. However, the application of posterior and anterior instrumentation with autologous grafts for spinal TB has been reported to be superior to local debridement and anterior bone grafting in recent studies^4–6^. Autologous iliac crest grafts have long been considered the gold standard grafts for anterior interbody fusion in the treatment of spinal TB, but donor site complications limit autograft availability and lead surgeons to choose effective graft alternatives. With the development of allograft processing and sterilization techniques guided by the American Association of Tissue Banks (AATB) standards, bone allografts have been widely used in spine surgery for several decades^7,8^. Some pioneers have used fresh-frozen femoral or humeral rings to perform interbody fusion in spinal TB patients and found that allografts and anterior instrumentation are effective in supporting the anterior spinal column^9–11^. Some people are concerned about the risk of M. TB persisting and recurring if devitalized bone grafts are used, so the use of bone allografts for the treatment of spinal TB remains controversial.

In this study, the effectiveness of single-stage surgery for spinal TB, which involved interbody fusion with freeze-dried tricortical iliac crest allografts and anterolateral single rod instrumentation, was assessed by the intra- and post-operative outcomes.

## Results

The average operation time was 203.66 ± 43.12 min (range 150–450 min). The mean blood loss was 530.45 ± 121.63 ml (range 200–1,000 ml), and an average of 2.31 ± 1.22 units of banked blood was transfused. All diagnoses were confirmed by histopathological examination.

In this series, there were no serious perioperative complications except pleura injury (one patient) or antiTB drug-induced hepatitis (one patient). No patients had superficial wound infections or sinus tract formation, and all the incisions healed within 2 weeks. Two patients had recurrence of psoas abscess because of inadequate debridement. Revision anterior debridement was performed for these patients.

The mean follow-up period was 37.5 months (range 24–120) for 52 patients, and 4 patients were lost to follow-up. Screw cutting of a single rod/screw system occurred in 8 patients (15.38%) with thoracic spine involvement. No graft fracture, collapse, or sliding was observed. The average time to bony fusion was 10.6 months (range 6–18 months). Bony fusion was observed in 8 patients at 6 months, 16 patients at 9 months, 17 patients at 12 months, and all 52 patients by 18 months. The average degrees of kyphotic correction loss in the thoracic and lumbar TB patients were 6.71° and 2.78°, respectively (Table 1).

**Table 1:** The average preoperative (PR) and postoperative (PO) local kyphosis (LK), values of correction loss (CL) and last follow-up (LF) of the patients according to vertebral regions.

	PRLK (°)	POLK (°)	LFLK (°)	CLLK (°)	p
Thoracic (n:39)	35.63 ± 11.66 (13–55)	16.93 ± 8.72 (10 to 37)	23.64 ± 11.73 (10 to 35)	6.71	< 0.05
Lumbar (n:13)	7.33 ± 4.23 (3–12)	− 6.23 ± − 3.14 (− 3 to − 12)	− 3.45 ± − 1.51 (− 3 to − 6)	2.78	< 0.05

*n* number of patient.

### Neurological function

Twenty-one of 24 patients showed total neurological function recovery (87.50%), one patient with severe spastic paraplegia did not show obvious recovery (ASIA score was C), one patient improved from ASIA score B–D, and one patient whose preoperative ASIA score was a D showed slight improvement, but the score remained a D. It took 7 days to 6 months for the patients with radical decompression to recover, and the mean times required for recovery for patients with preoperative ASIA scores B, C, and D were 98.3 days, 48.5 days, and 35.7 days, respectively.

## Discussion

The Medical Research Council Working Party on TB of the Spine recommends conservative chemotherapy as the first-line treatment in developing countries, and in many cases of Pott’s disease, it has demonstrated satisfactory results^12,13^. Only by effective chemotherapy regimens can the mortality associated with perioperative TB diffusion be minimized. We considered preoperative chemotherapy effective when patients’ weakness and anorexia symptoms improved and the ESR level decreased to 50 mm/h. To improve patient compliance, short-course chemotherapy with or without radical surgery has been studied, but to date, there are no strong evidence-based medicine guidelines for short-course chemotherapy for osteoarticular TB. We have used the 18-month four drug regimens for decades with satisfactory outcomes.

Doctors should be aware that the goals for spinal TB management include but are not limited to eradicating the tuberculous infection and reliving pain. Destruction of the anterior and middle spinal columns leads to biomechanical instability, and persistent anterior compression may cause irreversible neurological dysfunction. For some patients, radical surgery may be the most effective treatment to shorten the duration of the disease, restore spinal stability, and preserve and restore neurological function. The radical Hong Kong procedure introduced by Hodgson and Stock has long been considered the gold standard for the surgical management of spinal TB. Although it has been shown to be superior to local debridement in preventing early and late deformities, the procedure still has pitfalls in correcting deformities and preventing the progression of kyphosis, and the rate of bone graft failure remains high^14^. Moreover, a long period of complete bed rest causes considerable patient inconvenience, and much effort from family members is also needed for postoperative nursing in China. The need for more rapid cures has been recognized, and many attempts have been made to meet this need. In the last two decades, the use of posterior and anterior instrumentation with anterior interbody fusion in spinal TB patients has been reported to be successful and provide stability. Obviously, ambulant treatment leads to rapid recovery, and early mobilization and can prevent complications associated with prolonged bed rest and improve patients' quality of life.

The correct use and selection of spinal implant types are important to obtain satisfactory results in spinal TB surgery. There is a risk of persistent tuberculous infection associated with internal fixation, so anterior grafting with posterior instrumentation has been recommended for the stabilization of the involved motion segments and the correction of deformities. However, complications related to the posterior procedure cannot be ignored^15^. The simpler the procedure is, the better the outcomes. Currently, single-stage anterior surgery for spinal TB is used, and it has been indicated to be effective for select patients. Our results suggest that anterior interbody fusion and anterolateral single rod instrumentation provide the following advantages: short fusion and fixation segments, no injury to the posterior column, a short operative time, a small volume of blood loss, the maintenance of proper correction, immediate rigid stability, and low costs^16–19^.

Most of the patients in our study are low-income residents, so a lower cost and more effective management are expected. Surgeons must balance medical expenses and the effect of internal instrumentation. Therefore, an anterolateral single rod system was used in the thoracic and selected lumbar spine. The rib cage can provide additional stability, and single stage anterior interbody fusion with anterolateral single rod instrumentation is possibly effective. In our series, satisfactory outcomes were obtained in 84.6% (44/52) of patients. Screw cuts occurred in 15.38% of patients with thoracic vertebrae involved, but bone fusions were finally achieved. The presence of screw cuts indicated that the stability of the rib cage could not be increased, and external support was necessary. Otherwise, the addition of a second rod may help partially control the flexion. For lumbar spinal TB cases, severe kyphosis and severe bone destruction were not observed. With immobilization with an orthosis, we encourage patients to perform early functional exercise so that rehabilitation is easy and quick.

Internal instrumentation can only provide temporary fixation, and the differences in segmental stability and kyphotic deformity correction loss have been linked to the type of bone graft material used. There are several options available for reconstructing structural voids in the spinal column after spinal debridement and decompression. Autograft bone, the gold standard bone graft material, show optimum skeletal integration, but the presence of host morbidity and donor site complications limit their availability^20^. With the development of allograft processing and sterilization techniques guided by AATB standards, bone allografts have been widely used in spine surgery for several decades. Experimental studies have confirmed an immune response to bone allografts, but their clinical significance in humans remains unclear^21,22^. Some pioneers have used fresh-frozen bone allografts to perform interbody fusion in spinal TB patients and found that allografts and anterior instrumentation are effective in supporting the anterior spinal column, and although fusion occurred late, the grafts remained stable^22,23^.

To repair post-debridement defects in upright human spines, the critical property of structural allografts is their compressive strength. Tricortical iliac crest allografts have acceptable strength to resist axial compression. For tricortical iliac grafts, the cortical portion of the graft provides structural support due to its high mechanical strength, while the cancellous portion provides a favourable environment for vascular ingrowth. More importantly, cancellous bone contact prevents the graft from sinking into the vertebrae, so the use of an iliac wedge does not require the availability of solid endplates of adjacent vertebrae. Stability can be strengthened when the wedge is used in combination with anterior instrumentation.

However, allograft bone transplantation also has disadvantages. For example, it can induce infectious diseases, but the risks can be minimized under strict procedures performed in accordance with the AATB standards. Slow bone integration is another problem that should be considered. Iliac crest allografts yield no osteoinductive effects, but osteoinduction may occur when the allografts are combined with Bone Morphogenetic Protein. The acellular and porous cancellous parts are associated with good osteoconductive effects. The morselized autologous grafts in the anterior one-third of the interbody space can produce a bony bridge as a sentinel sign. Although integration occurred later for the allograft iliac crest than for the autologous iliac crest, most clinicians will choose asymptomatic late fusion over a high rate of chronic pain in the donor site.

Paraplegia caused by spinal TB is the absolute indication to perform radical surgery. With the support of structural bone grafts and anterior instrumentation, radical debridement and decompression can be performed adequately. For most patients with Pott's paraplegia, compression occurred gradually, and the prognoses differed drastically from those of patients with traumatic paraplegia. Performing urgent decompression at the onset of the disease has relatively few adverse effects. However, severe spastic paraplegia that occurs during healing is difficult to treat, and it takes much more time to resolve; there are even some patients who do not recover.

## Conclusion

We believe that single-stage anterior interbody fusion combined with anterior instrumentation is safe and effective for most patients with thoracic and lumbar spine TB. Although the time required for complete integration was longer for tricortical iliac crest allografts than for the autografts, tricortical iliac crest allografts were found to be convenient and safe for use as bone graft materials in spinal TB surgery. They may be effective for interbody fusion and deformity correction if combined with anterior instrumentation.

## Methods

Between February 2004 and July 2018, 41 patients with thoracic spinal TB and 15 patients with lumbar spinal TB underwent single-stage surgery at our unit, which included anterior radical debridement, interbody fusion with tricortical iliac crest allografts and anterolateral single rod instrumentation. The research was approved by the Ethics Committee of the Southwest Hospital and all methods were performed in accordance with relevant regulations. All participants have been informed and gave written consent prior to data collection. Indications for this surgery included vertebral collapse and spinal instability, progressive kyphotic deformity and neurological involvement. Inclusion criteria included: severe back pain and/or radicular pain resistant to conservative treatment, cases with a kyphotic angle < 60°, lesions were main in the anterior and/or middle column, and the lesion were confined to less than three adjacent segments. Inclusion criteria included: the number of damaged vertebrae was more than 3, cases with cardiac-pulmonary morbidity, poor health of abdomen, cases with a kyphotic angle ≥ 60°. There were 29 males and 27 females, with a mean age of 31.7 years old (ranging 20–72 years).

### Preoperative evaluation and preparation

All patients were assessed clinically and radiologically following hospitalization. Almost all patients had general TB symptoms such as weight loss, night sweats, fatigue and predominantly back pain in their histories. In general, these patients were notable for having mild increases in the ESR level. At the time of presentation, six patients (10.71%) had active pulmonary system TB, twenty-four patients (42.85%) presented with partial or complete neural deficits, and neurological function was graded according to the American Spinal Injury Association (ASIA) scoring system.

The roentgenograms revealed narrow disc spaces, paravertebral abscess formation, and kyphotic deformities due to vertebral collapse. Two contiguous vertebrae were involved in 40 cases, and three vertebrae were involved in 16 cases. Local kyphosis was measured as the angle between the upper and lower end plates of the collapsed levels. The average degree of kyphotic deformity in the patients with thoracic and lumbar TB was 35.63 ± 11.66° (range 13°–55°) and 7.33° ± 4.23° (range 3°–12°), respectively (Table 1). All patients were evaluated with computerized tomography (CT) and magnetic resonance imaging (MRI).

All patients, with the exception of those who had progressive neurologic deficits that necessitated urgent decompression, underwent antituberculosis chemotherapy prior to surgery for at least two weeks. The chemotherapy regimen consisted of rifampicin (R) 15 mg/kg (maximum 600 mg/day), isoniazid (INH) 6 mg/kg (maximum 300 mg/day), ethambutol (EMB) 25 mg/kg/day (maximum 2.5 g/day), and pyrazinamide (PZA) 30 mg/kg (maximum 60 mg/kg)^12^.

All the patients were made fully aware of the risks, benefits, and alternatives to the management and signed the informed consent form for the use of allograft bone.

### Operative method

**Figure 1:**
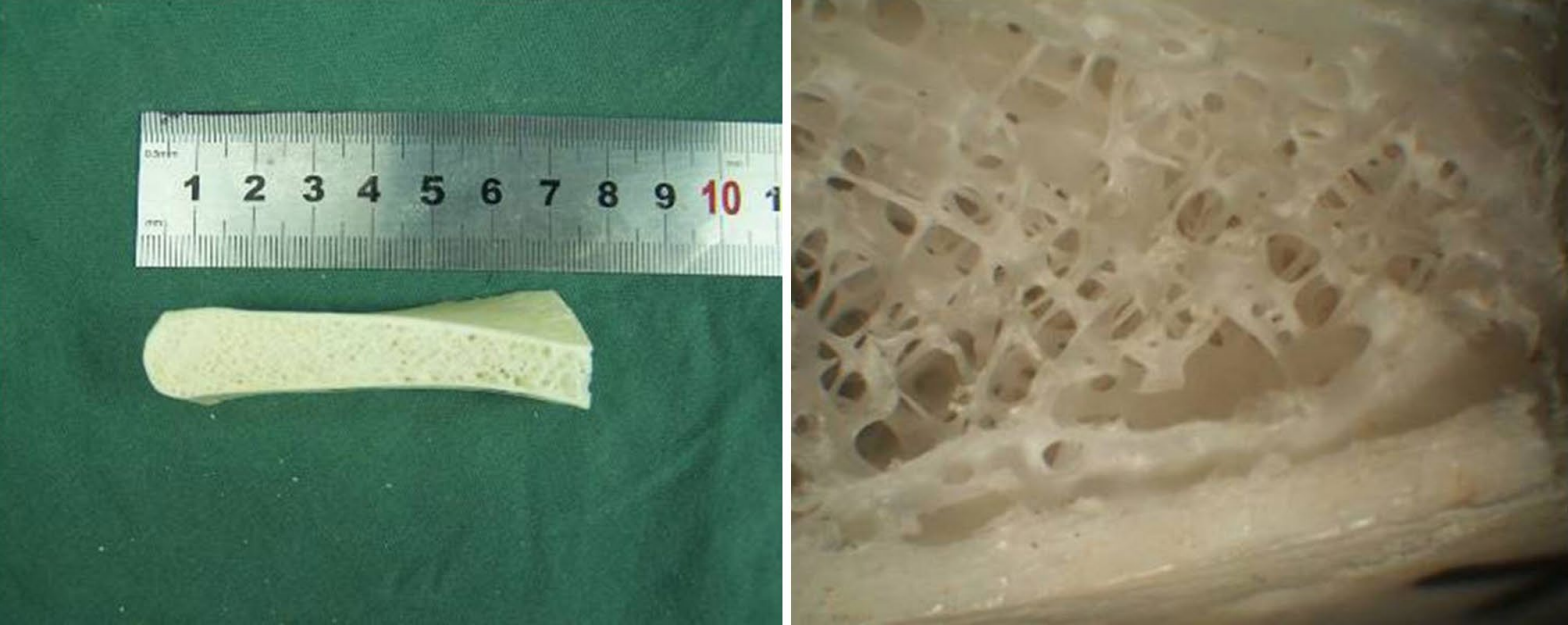
Freeze-dried tricortical iliac crest allograft wedge without bone marrow cells.

The involved vertebrae were accessed by thoracotomy in the thoracic region and thoracophrenolumbotomy in the lumbar region. If an abscess was present, it was drained first, and neural decompression was done with complete corpectomy of the destroyed vertebra. After radical debridement, a proper heighten freeze-dried tricortical iliac crest allograft from our hospital bone bank was placed into the defect to reconstruct the anterior column, and fragments of the rib were planted into the void in front of the allograft (Fig. 1).

**Figure 2:**
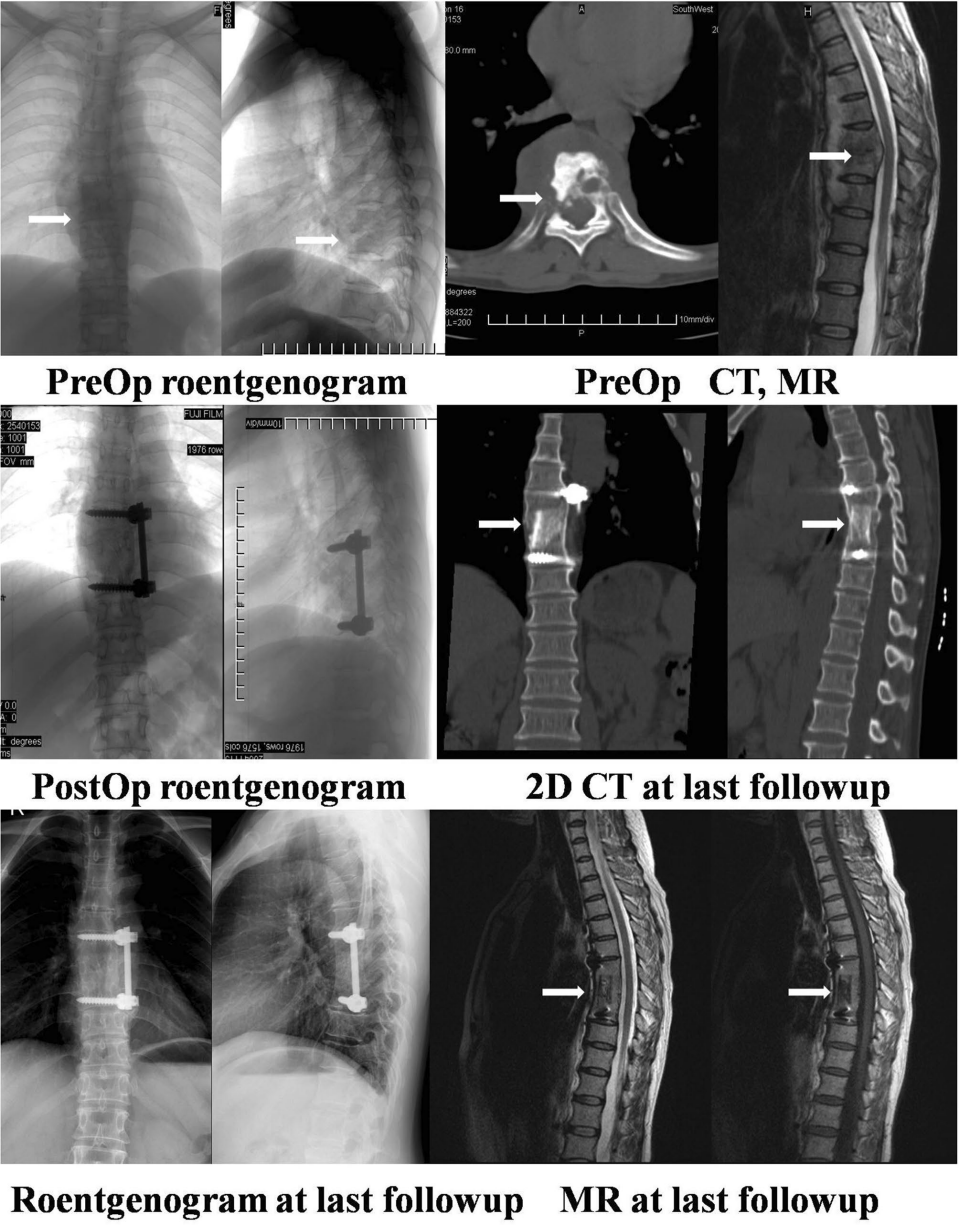
A 35-year-old woman complained of severe back pain and lower extremity weakness. Preoperative radiographs, CT scans and MRI scans showed T8/9 disc space with the destruction of adjacent vertebral bodies and paravertebral abscess with spinal cord compression (white arrows). The kyphotic angle was 15°. The anteroposterior and lateral roentgenograms taken after surgery showed the long segment tricortical iliac crest allograft inserted from T7–T9. The CDH rod was placed for added stability to prevent graft displacement. The kyphotic angle improved to 9°. The 2D CT scan taken at the 10-year follow-up showed that the graft integrated with the adjacent vertebral bodies. The roentgenogram showed solid bony fusion, and the loss of correction was 3°. The MR scan taken at the final follow-up showed healed vertebrae and no compression of the spinal cord.

**Figure 3:**
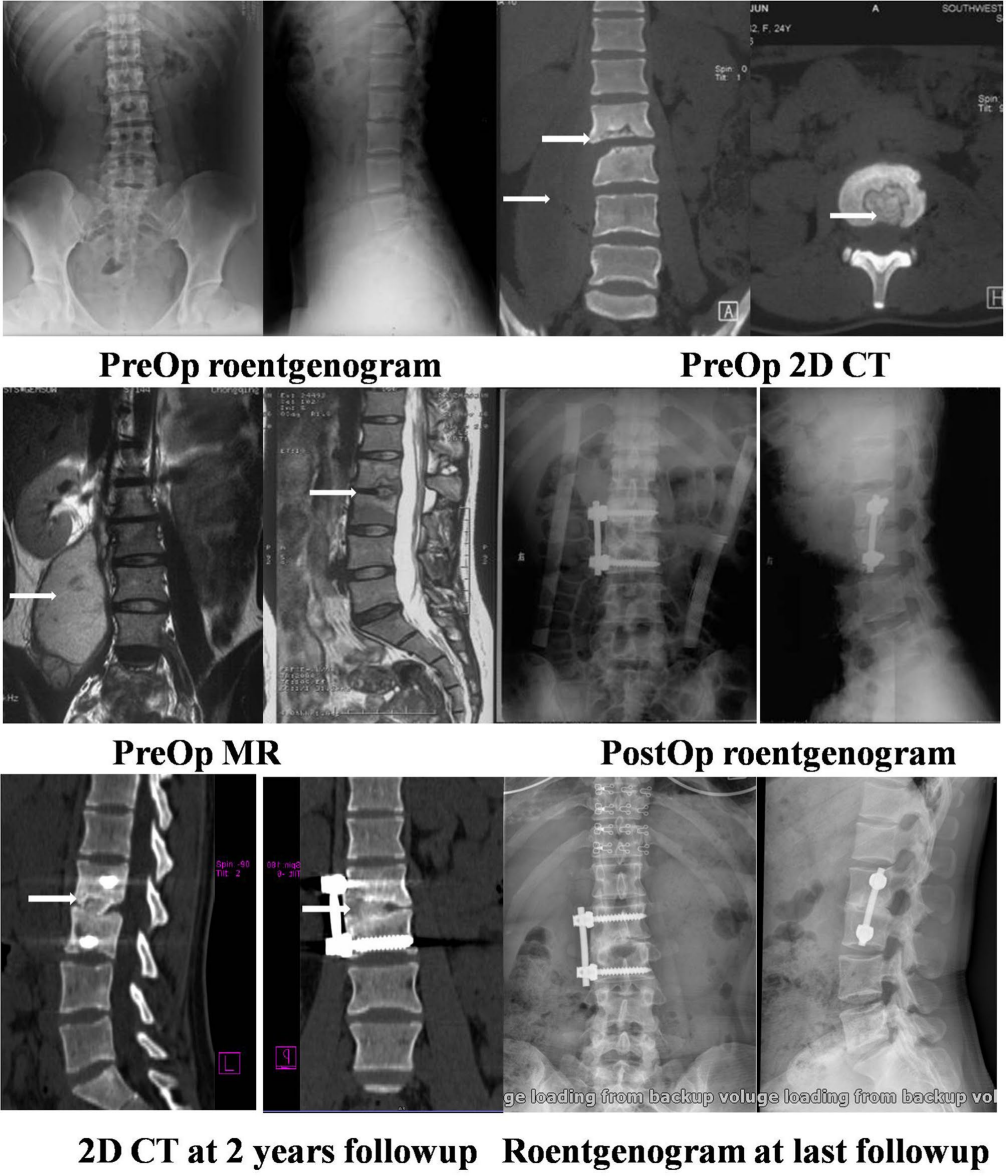
A 25-year-old woman complained of severe back pain. The preoperative roentgenogram, 2D CT scan and MRI scan showed destruction of L2/3 vertebral bodies and right psoas abscess (white arrows). The immediate postoperative roentgenogram showed the tricortical iliac crest allograft and anterolateral single rod instrumentation. The 2D CT scan taken at the 2-year follow-up showed good incorporation and normal lordosis. The roentgenogram taken at the 9-year follow-up showed that solid bony fusion was achieved, and the loss of correction was 4°.

The rod screw systems were placed by experienced spinal surgeons with specific training in the use of these systems. Titanium A titanium pedicle screws were placed into the vertebrae just above and below the fused segments, then a single rod implant apparatus was assembled. Importantly, in order to shorten the segment of fusion, the TB-affected vertebrae were prior to choose, if its remained height above second of third of normal. Anterior instrumentation was performed using the titanium Cotrel-Dubousset-Hopf (CDH) method in all patients. Finally, 0.6 g isoniazid and 1 g streptomycin were injected into the focus of the lesion, and the wound was closed routinely. Catheter drainage was performed in all patients (Figs. 2, 3).

### Postoperative management

Nutrition support and 18 months of standard chemotherapy were administered to all patients^24^. Patients were encouraged to stand and walk on the fifth postoperative day. Active rehabilitation was started immediately for the patients who had neural deficits. All patients were followed by immobilization in a brace for 6 months. The patients were followed up a month later, every 3 months for the following 12 months, and then at intervals of half a year; the clinical status, roentgenogram results, ESR levels and liver function test results were the outcome measures. The radiographs were analysed before surgery, immediately after surgery, and at the last follow-up to determine the success of anterior fusion and maintenance of correction.

The presence of significant consolidation, along with the absence of implant failure or correction loss and pain relief, was considered a sign of fusion. At the last follow-up, implant failure and other complications were also evaluated.

Statistical analyses were performed using SPSS version 22 (SPSS, Inc., Chicago, USA). Tests used for statistical analyses included the Wilcoxon matched-pair signed-rank test and paired samples t-test, with a confidence interval of 95%.

### Ethical approval

This research was approved by the Ethics Committee of Southwest hospital, the First Affiliated Hospital of the Third Military Medical University, People's Liberation Army (PLA). All participants have been informed and gave written consent prior to data collection.

### Consent for publication

Written informed consent was obtained from the patients for publication of their clinical details and clinical images.

## Data Availability

The datasets used and analysed during the current study are available from the corresponding author upon reasonable request.

## Abbreviations

TB: Tuberculosis
ASIA: American Spinal Injury Association
ESR: Erythrocyte sedimentation rate
HREZ: Isoniazid, rifampicin, ethambutol, and pyrazinamide
VAS: Visual analogue score
